# Identification of MicroRNAs as Potential Blood-Based Biomarkers for Diagnosis and Therapeutic Monitoring of Active Tuberculosis

**DOI:** 10.3390/diagnostics12020369

**Published:** 2022-02-01

**Authors:** Junseong Kim, Heechul Park, Sung-Bae Park, Eun Ju Lee, Min-A Je, Eunsol Ahn, Bora Sim, Jiyoung Lee, Hyunwoo Jin, Kyung Eun Lee, Sang-Nae Cho, Young Ae Kang, Hyejon Lee, Sunghyun Kim, Jungho Kim

**Affiliations:** 1 Department of Clinical Laboratory Science, College of Health Sciences, Catholic University of Pusan, Busan 46252, Korea; wnstjd028@naver.com (J.K.); wt2626@naver.com (H.P.); tjdqo2013@naver.com (S.-B.P.); leeeunju41@naver.com (E.J.L.); je.mina0504@gmail.com (M.-A.J.); lab84@hanmail.net (J.L.); jjinhw@cup.ac.kr (H.J.); kelee@cup.ac.kr (K.E.L.); 2 Clinical Trial Specialist Program for In Vitro Diagnostics, Brain Busan 21 Plus Program, Graduate School, Catholic University of Pusan, Busan 46252, Korea; 3 Clinical Vaccine Research Section, International Tuberculosis Research Center, Seoul 03772, Korea; eunsol486@naver.com (E.A.); RAYCHO@yuhs.ac (S.-N.C.); 4 Department of Microbiology, Institute of Immunology and Immunological Disease, Yonsei University College of Medicine, Seoul 03772, Korea; SIMBR0129@yuhs.ac; 5 Division of Pulmonary and Critical Care Medicine, Department of Internal Medicine, Severance Hospital, Institute of Immunology and Immunological Disease, Yonsei University College of Medicine, Seoul 03772, Korea; MDKANG@yuhs.ac

**Keywords:** tuberculosis, latent tuberculosis infection, biomarkers, microRNAs

## Abstract

Early diagnosis increases the treatment success rate for active tuberculosis (ATB) and decreases mortality. MicroRNAs (miRNAs) have been studied as blood-based markers of several infectious diseases. We performed miRNA profiling to identify differentially expressed (DE) miRNAs using whole blood samples from 10 healthy controls (HCs), 15 subjects with latent tuberculosis infection (LTBI), and 12 patients with ATB, and investigated the expression of the top six miRNAs at diagnosis and over the treatment period in addition to performing miRNA-target gene network and gene ontology analyses. miRNA profiling identified 84 DE miRNAs in patients with ATB, including 80 upregulated and four downregulated miRNAs. Receiver operating characteristic curves of the top six miRNAs exhibited excellent distinguishing efficiency with an area under curve (AUC) value > 0.85. Among them, miR-199a-3p and miR-6886-3p can differentiate between ATB and LTBI. Anti-TB treatment restored the levels of miR-199b-3p, miR-199a-3p, miR-16-5p, and miR-374c-5p to HC levels. Furthermore, 108 predicted target genes were related to the regulation of cellular amide metabolism, intrinsic apoptotic signaling, translation, transforming growth factor beta receptor signaling, and cysteine-type endopeptidase activity. The DE miRNAs identified herein are potential biomarkers for diagnosis and therapeutic monitoring in ATB.

## 1. Introduction

Tuberculosis (TB) is a fatal infectious disease caused by *Mycobacterium tuberculosis* (MTB) infection [1]. According to the World Health Organization (WHO), there were approximately 10 million new cases and 1.2 million deaths attributed to TB worldwide in 2019 [2]. Approximately 85% of MTB infections occur in the lungs but infections can spread and affect multiple organs [1]. According to a WHO report in 2021, TB is the most powerful single pathogen to have emerged before coronavirus disease 2019 (COVID-19) and about one-quarter of the world’s population is infected with MTB. Of all patients infected with MTB, about 5% of them will have active tuberculosis (ATB), and about 95% will develop latent TB infection (LTBI). It is likely that they will develop ATB during their lifetime [3].

Early diagnosis and proper therapy can improve the outcomes of patients with ATB. Great advances and efforts have been made in recent years to treat and understand the basal mechanisms of MTB infection [4,5,6]. However, remission rates remain suboptimal, and TB diagnosis remains challenging, owing to the limitations in the specificity and sensitivity of the current diagnostic tests [7]. There are two typical methods for diagnosing latent MTB infection. The first is the tuberculin skin test (TST), which entails checking the skin reaction to injected antigen, but the reaction is influenced by Bacillus Calmette-Guérin (BCG) vaccination status and non-tuberculous mycobacterial infection, often yielding false results [8]. The second is a commercial interferon gamma (IFN-γ) release assay called the QuantiFERON-TB gold in-tube (QFT-GIT) test method, which is faster and more sensitive than TST but cannot differentiate between patients with ATB and LTBI [9]. Thus, there is a need for a rapid method that accurately distinguishes the disease state with high sensitivity and specificity.

There is a growing interest in microRNAs (miRNAs) as biomarkers of various diseases. miRNAs are short (approximately 22 nucleotides) non-coding RNAs that bind to RNA sequences and perform post-transcriptional gene silencing [10]. When miRNA binds to a complementary sequence in an mRNA molecule, it cuts the mRNA strand, destabilizes the mRNA by shortening the poly(A) tail, or renders the translation of mRNA into protein by ribosomes less efficient, resulting in gene silencing [11,12]. Moreover, miRNAs are involved in the pathogenesis and progression of a wide range of infectious disease processes, including regulating mRNA expression, which modulates innate and adaptive immune responses [13]. Numerous studies have confirmed that miRNAs play important roles in the pathogenesis and progression of TB, and the diagnostic potential of serum miRNA levels has motivated researchers to evaluate their clinical utility in several diseases, including TB and nontuberculous mycobacterial infections [14,15]. Some studies have reported that the host immune response to MTB is modulated by specific miRNAs, and that most are induced by MTB infection [16,17,18,19,20,21,22]. Of these, miR-125b and miR-155 were shown to affect an inflammatory response against MTB infection by targeting TNF-α and SHIP1. Moreover, distinct macrophage miRNA expression was elicited according to the virulence of MTB, suggesting an important role for miRNAs in pathogenesis [16,18,23]. Although the function of miRNAs in TB has been established, the potential diagnostic or prognostic value of miRNAs in clinical settings has not been fully investigated.

The effective discovery of diagnostic biomarkers and therapeutic targets has been emerging with the advances in genetic analysis techniques and bioinformatics analysis [24]. Furthermore, miRNAs function in the regulation of multiple genes and modulate activators or suppressors of signaling networks in biological processes [25]. Thus, a comprehensive analysis of miRNAs and the identification of key target genes can provide pivotal opportunities for disease diagnosis and treatment. Herein, we aimed to identify clinically usable differentially expressed (DE) miRNAs using whole blood samples from 10 healthy controls (HCs), 15 individuals with LTBI, and 12 patients with ATB, in order to test the hypothesis that levels of DE miRNAs change over the course of anti-TB drug therapy in patients with ATB. The diagnostic value of DE miRNAs was assessed in terms of sensitivity, specificity, and the area under the curve (AUC) of the receiver operating characteristic (ROC) curve. Finally, we explored the target genes predicted by miRNet to determine their potential roles in TB pathogenesis and disease progression using pathway analysis.

## 2. Materials and Methods

### 2.1. Clinical Samples

Blood samples were collected from Yonsei University Severance Hospital, a tertiary referral hospital in Seoul, Republic of Korea, between January 2016 and September 2019. The study was approved by the Institutional Ethics Committee of Yonsei University Severance Hospital (IRB No. 4-2014-1108), and written informed consent was obtained from all participants for the identification of TB biomarkers. Individuals with any acute or chronic disease, previous history of ATB, or symptoms suggestive of TB were excluded. We classified the study participants into an ATB group, an LTBI group, and a healthy control (HC) group [26,27].

1. ATB: patients with ATB were diagnosed based on microbiological and pathological data. A positive MTB culture result from respiratory specimens or the presence of caseous granulomas in lung tissue was used to confirm the diagnosis of ATB. Cases of clinical ATB, which was defined as negative mycobacterial culture findings but favorable clinical and radiological response to anti-TB medication, were also included.
2. LTBI: recent contact with ATB patients testing positive for the QFT-GIT were enrolled. The participants had no history of TB and no suggestive symptoms of TB.
3. HC: the HCs had to be TB-negative based on the QFT-GIT assay and chest x-rays, and no history of TB. They were free of ATB symptoms, and had no recent contact with patients with ATB.

### 2.2. AFB Smear and Culture

For acid-fast bacillus (AFB) detection, auramine–rhodamine fluorescent staining was performed using sputum samples, and the results were confirmed using the Ziehl–Neelsen method [28,29]. Mycobacterial culture was conducted using liquid and solid culture media, and the *M. tuberculosis* complex was confirmed by a duplex PCR using Advansure TB/NTM real-time PCR (LG chemistry, Seoul, Korea). The mycobacterium culture and AFB smear of sputum samples were conducted in a biosafety cabinet class 2, in compliance with the local and national regulations on containment equipment.

### 2.3. QFT-GIT

The QFT-GIT test, a commercial TB-specific interferon gamma release assay (QIAGEN, Hilden, Germany), was performed according to the manufacturer’s instructions. Briefly, 1 mL of whole blood was collected in three QFT-GIT collection tubes for no stimulation (negative control), MTB-specific peptide stimulation (ESAT-6, CFP-10, and TB7.7), and mitogen stimulation (positive control), and incubated for 16–24 h at 37 °C. Plasma was harvested by centrifugation at 3000× *g* for 15 min and stored at −80 °C until use. The concentration of IFN-γ was determined using a QFT-GIT enzyme-linked immunosorbent assay kit according to the manufacturer’s instructions. The QFT-GIT test results were interpreted using QFT-GIT software version 2.62 (QIAGEN).

### 2.4. miRNA Extraction

Three milliliters of whole blood were collected into PAXgene Blood RNA tubes (QIAGEN) at diagnosis (T0), at 2 months (T2; during treatment), and at 6–9 months after treatment (T6; completion of treatment), and stored at −80 °C. miRNAs were extracted using the PAXgene Blood miRNA kit (QIAGEN) according to the manufacturer’s instructions. The RNA concentration was measured using a NanoDrop spectrophotometer (Thermo Fisher Scientific, Waltham, MA, USA). The RNA integrity number (RIN) was determined using a BioAnalyzer 2100 (Agilent, Santa Clara, CA, USA), and sequencing was performed using only samples with RIN ≥ 7.0. The RNA samples were stored at −80 °C until use.

### 2.5. miRNA Sequence Analysis

Small RNA libraries were prepared using the NEBNext Multiplex Small RNA Library Prep kit (Illumina, San Diego, CA, USA) according to the manufacturer’s instructions. Briefly, an RNA sequencing library was generated on the Illumina NextSeq500 platform via cDNA amplification, end-repair, adenylation of 3′ ends, adapter ligation, and amplification.

### 2.6. miRNA Expression Profiles

The miRNAs obtained via sequencing were analyzed using MultiExperiment Viewer (MeV 4.9.0; The Perl Foundation, Holland, MI, USA). Hierarchical clustering and volcano plot analyses were performed to identify DE miRNAs. DE miRNAs were identified using cut-off points for the *p* value (<0.05; −log_10_(0.05) = 1.30) and mean difference (>0.5 or <−0.5).

### 2.7. qRT-PCR Validation

To analyze the expression of DE miRNAs (miR-16-5p, miR-199-3p (detection of both miR-199a-3p and miR-199b-3p), miR-374c-5p, miR-6886-3p, and miR-6856-3p), blood samples after MTB-specific peptide stimulation were treated using 500 µL of RNA/DNA stabilization reagent for blood/bone marrow (Roche Diagnostics, Mannheim, Germany). Subsequently, a MagNA Pure LC RNA Isolation Kit-High Performance kit (Roche Diagnostics) was used to extract RNA according to manufacturer’s instructions [30].

Next, complementary DNA (cDNA) of miR-16-5p, miR-199a-3p, miR-199b-3p, miR-374c-5p, miR-6886-3p, and miR-6856-3p was synthesized using the miRCURY LNA RT Kit (Qiagen) following manufacturer’s protocols. The temperature profile for cDNA synthesis reaction was as follows: 42 °C for 60 min and 95 °C for 5 min.

Quantitative reverse transcriptase (qRT)-PCR was performed using the miRCURY LNA miRNA PCR Assay [31]. PCR cycling was performed as follows: 95 °C for 2 min, followed by 40 cycles of 95 °C for 10 s and 56 °C for 1 min. qRT-PCR were performed on the CFX96 Real-time PCR System Detector (Bio-Rad, Hercules, CA, USA). Samples were run in duplicate for each experiment. To monitor contamination of the reagents, a negative control was included for each primer pair. Data were analyzed using the comparative ΔC_T_ method (2^−ΔCT^), with RNU44 as an endogenous control [32].

### 2.8. miRNA-Target Gene Network Construction and Gene Ontology (GO) Enrichment Analysis

The web-based platform miRNet (http://www.mirnet.ca/, accessed on 17 December 2021) was used to predict genes targeted by the selected DE miRNAs. The miRNA target gene network was constructed based on the mapping analysis. For GO enrichment analysis, the target genes in the network were analyzed using Cytoscape Version 3.8.1 (Cytoscape Team, Seattle, WA, USA) with ClueGO. ClueGO parameters were set as follows: GO term fusion was selected including the biological process, cellular component, and molecular function; only pathways with *p* < 0.05 in Bonferroni step-down analysis were included; kappa score of 0.4 [33].

### 2.9. Statistical Analysis

The data were statistically analyzed using IBM SPSS Statistics for Windows, version 21.0 (IBM, Armonk, NY, USA), GraphPad Prism software Version 6.0 (GraphPad, La Jolla, CA, USA) and MeV 4.9.0 (The Perl Foundation). Comparisons for each group were conducted using the Mann–Whitney *U* test or one-way analysis of variance test. ROC curve analysis was performed to evaluate the discriminating factors of miRNAs. To assess the diagnostic utility of the DE miRNAs, we investigated the AUC values of the ROC curve, as well as statistical sensitivity and specificity. Statistical significance was set at *p* < 0.05.

## 3. Results

### 3.1. Characteristics of Study Participants

A total of 37 study participants including 12 with ATB, 15 with LTBI, and 10 HCs were recruited (Table 1). The age (mean ± standard deviation) of the ATB, LTBI, and HC groups were 41.0 ± 19.6 years, 42.1 ± 15.1 years, and 34.4 ± 7.2 years, respectively. The percentages of male patients in the ATB, LTBI, and HC groups were 66.7% (8/12), 40.0% (6/15), and 60% (6/10), respectively. BCG scars were present in 9 (75.0%) patients with ATB and 8 (53.3%) patients with LTBI, and in 9 (90.0%) HCs. There were no significant differences in demographic characteristics among the three groups.

### 3.2. Identification of Potential miRNAs as Diagnostic Biomarker for TB

**Figure 1:**
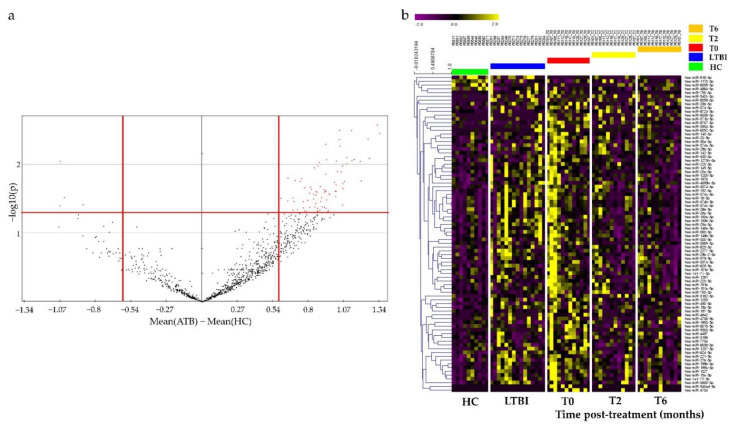
Volcano plot and heat map of differentially expressed miRNAs in whole blood of patients with active tuberculosis (ATB), latent tuberculosis infection (LTBI) and healthy controls (HCs). (**a**) Volcano plot of miRNAs between patients with ATB and HCs. Cut-off points for the *p* value (<0.05; −log_10_(0.05) = 1.30) or mean difference (>0.5 or <−0.5) are indicated by red lines. (**b**) Heat map of the 84 significantly differentially expressed miRNAs for patients with ATB after two (T2) and six months of treatment (T6), LTBI, and HCs (Pearson correlation; *p* < 0.05 by hierarchical clustering analysis). Yellow dots represent upregulated miRNAs, and blue dots represent downregulated miRNAs.

**Figure 2:**
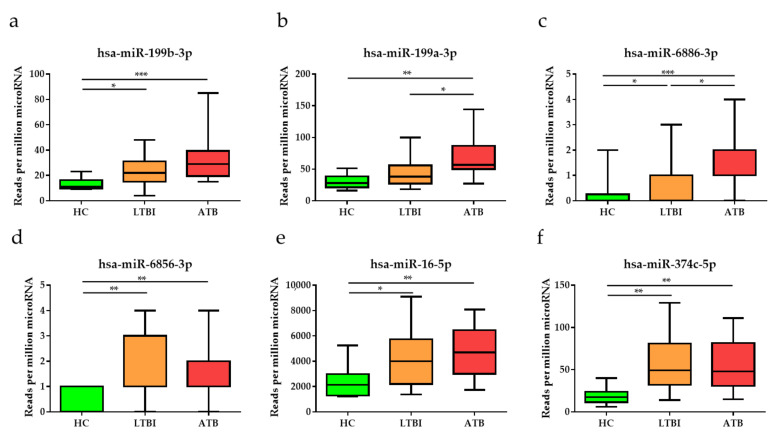
Expression levels of the top six differentially expressed miRNAs, namely (**a**) miR-199b-3p, (**b**) miR-199a-3p, (**c**) miR-6886-3p, (**d**) miR-6856-3p, (**e**) miR-16-5p, and (**f**) miR-374c-5p, in patients with active tuberculosis (ATB), individuals with latent tuberculosis infection (LTBI), and healthy controls (HCs). Data are reported as mean ± standard deviation. * *p* < 0.05, ** *p* < 0.01, *** *p* < 0.001.

To identify miRNAs for potential use as diagnostic biomarkers for TB, miRNA sequencing was performed using total RNA extracted from the blood of study participants, including a complete set of serial samples (T0, T2, and T6) from patients with ATB who underwent a six-month course of treatment. Potential DE miRNAs obtained via miRNA sequencing were selected based on specific criteria (mean difference >0.5 or <−0.5; *p* < 0.05), and 84 DE miRNAs, including 80 upregulated and 4 downregulated miRNAs, were identified via volcano plot analysis (Figure 1a). A heat map was generated to classify 84 miRNAs according to their infection status and treatment status (Figure 1b). As the disease progressed (HC to LTBI to ATB), the expression levels of various miRNAs clearly increased or decreased. The top six among them, namely miR-199b-3p, miR-199a-3p, miR-6886-3p, miR-6856-3p, miR-16-5p, and miR-374c-5p, tended to increase as the infection progressed (Figure 2). To validate and confirm whether DE miRNAs are specific to TB, we further performed qRT-PCR verification of the top six DE miRNAs using 51 additional MTB-specific peptide stimulation blood samples (17 HC, 22 LTBI, and 12 ATB samples) (Table S1). miR-199a/b-3p, miR-6856-3p, miR-16-5p and miR-374c-5p were observed to be highly expressed in TB samples (Figure S1).

### 3.3. Diagnostic Performance of the Top Six DE miRNAs

To investigate the clinical relevance of the top six DE miRNAs, the AUC, sensitivity, and specificity of miR-199b-3p, miR-199a-3p, miR-6886-3p, miR-6856-3p, miR-16-5p, and miR-374c-5p were analyzed (Table 2). All had AUC values of 0.86 or higher, indicating good diagnostic performance in ATB (*p* < 0.001). In particular, miR-199b-3p showed high sensitivity of 91.67% and specificity of 90.00%. Table S2 lists the positive and negative results for the top six DE miRNAs along with the coincidence rate with the culture results.

### 3.4. Anti-TB Treatment Response Profiles of Top Six DE miRNAs

**Figure 3:**
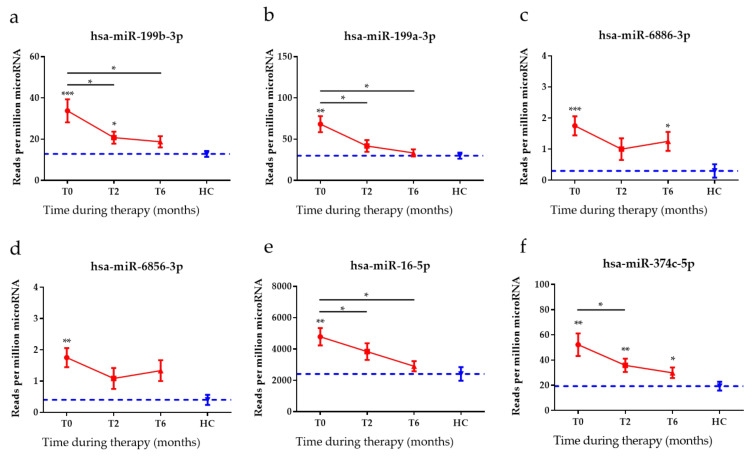
The expression levels of the top six miRNAs in patients with active tuberculosis (ATB) before, during and after treatment. The expression levels of (**a**) miR-199b-3p, (**b**) miR-199a-3p, (**c**) miR-6886-3p, (**d**) miR-6856-3p, (**e**) miR-16-5p, and (**f**) miR-374c-5p were measured in patients with ATB collected at the time of diagnosis (T0), after two months of therapy (T2), and after the completion of therapy (T6; 6–9 months after T0). The blue dotted line is representative of the mean miRNA expression level of the healthy control (HC) patients. The data are shown as mean ± standard error of the mean. * *p* < 0.05, ** *p* < 0.001, *** *p* < 0.0001.

To identify the response to anti-TB therapy in patients with TB during their treatment course, the expression levels of the top six DE miRNAs were assessed after two months of treatment (T2) and again at the end of standard anti-TB therapy, six to nine months after diagnosis (T6). The expression levels of all of the top six DE miRNAs were diminished in patients with ATB after two months and at the end of treatment (Figure 3). In particular, miR-199b-3p, miR-199a-3p, and miR-16-5p levels significantly decreased within 2 months of therapy and continued to trend towards HC levels after completion of therapy.

### 3.5. Construction of miRNAs and Target Gene Networks and GO Analysis of Target Genes

**Figure 4:**
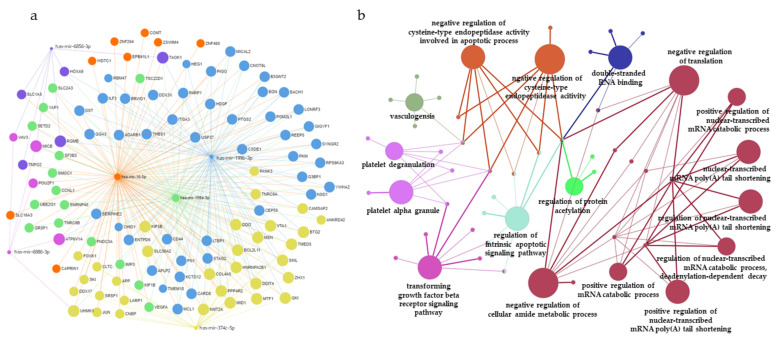
miRNA–mRNA interaction network analysis and gene ontology (GO) enrichment analysis of target genes. (**a**) The top six miRNAs were uploaded to the miRNet database and. A significant miRNA–target gene network was constructed; miR-199b-3p (blue), miR-16-5p (orange), miR-199a-3p (green), miR-374c-5p (yellow), miR-6856-3p (purple), and miR-6886-3p (pink). (**b**) GO enrichment analysis of target genes, with the top six differentially expressed miRNAs represented as functionally grouped networks of enriched GO terms generated by ClueGo. The parameters of ClueGO were set as follows: GO term fusion selected; only display GO terms with *p* < 0.05 in Bonferroni step-down analysis; kappa score of 0.4.

To further investigate the putative biological functions of the top six DE miRNAs, we predicted the target genes of each miRNA using miRNet (http://www.mirnet.ca/, accessed on 17 December 2021). In total, 108 predicted target genes were identified (Table S3). Figure 4a shows the miRNA-target gene network for the top six DE miRNAs.

Subsequently, GO analysis was performed to classify 108 target genes based on biological processes, molecular functions, and cellular components using the ClueGO plug-in of Cytoscape (kappa score = 0.4; *p* < 0.05, Bonferroni step-down analysis) (Figure 4b). The potential target genes were associated with negative regulation of cellular amide metabolic process (GO:0034249), followed by transforming growth factor beta receptor signaling pathway (GO:0007179), negative regulation of translation (GO:0017148), regulation of intrinsic apoptotic signaling pathway (GO:2001242), platelet degranulation (GO:0002576), platelet alpha granule (GO:0031091), and double-stranded RNA binding (GO:0003725). The GO analysis of the predicted targets is presented in Table 3 and Table S4.

## 4. Discussion

TB is one of the most important infectious diseases, with a high burden of morbidity and mortality [34,35]. Despite the studies aimed at diagnosis and therapy of disease, the development of new screening methods that can detect high-risk conditions and predict treatment responses with easy-to-manage assays is needed for the majority of infected subjects, especially in low-income countries [36]. Currently, many studies are being conducted to improve TB diagnostics, and in particular, diagnostic methods utilizing the immune system are attracting interest [37,38,39].

miRNAs, also known as epigenetic modulators, participate in many biological processes and pathogenic conditions by regulating post-transcriptional gene expression [40]. Recent studies have found that the expression of specific miRNAs differ according to TB infection status [41]. Studies have been conducted to elucidate the mechanism of miRNAs in several disease processes and whether they hold potential as diagnostic, prognostic, and therapeutic biomarkers [24,42,43]. Here, miRNA expression profiling was performed using 37 blood samples consisting of 12 ATB, 15 LTBI, and 10 HC samples to identify potential miRNAs for TB diagnosis and treatment monitoring. Furthermore, a regulatory network was constructed between the identified miRNAs and targets, in addition to a GO analysis of the predicted target genes. First, our miRNA profiling analysis of HC and ATB groups identified 84 significant DE miRNAs, including four downregulated miRNAs and 80 upregulated miRNAs. Among these, the top six significant DE miRNAs, namely miR-199b-3p, miR-199a-3p, miR-6886-3p, miR-6856-3p, miR-16-5p, and miR-374c-5p, were significantly upregulated in the blood of patients with ATB compared with HCs. In particular, miR-199a-3p and miR-6886-3p could be used as biomarkers to detect TB infection and to differentiate between ATB and LTBI (*p* < 0.05 and *p* < 0.05, respectively). Interestingly, miR-199b-3p, miR-199a-3p, and miR-16 are reportedly related to immune responses and infectious diseases, based on the results of previous studies. It was found that miR-199b-3p expression was increased in septic acute kidney injury models, and that miR-199b-3p binds to nuclear factor erythroid 2-related factor (NRF2), which is a transcription factor directly involved in the transcriptional activation of genes involved in cellular antioxidant responses that plays a central role in the pathogenesis of TB [44,45]. Furthermore, miR-199a-3p can mediate immune tolerance by regulating dendritic cells, leading to increased secretion of interleukin 10 as well as to the inhibition of the phosphatidylinositol 3-kinase/protein kinase B/nuclear factor kappa B pathway, and miR-16 was significantly upregulated in the serum of patients with TB compared with healthy controls [46,47]. In addition to miR-199b-3p and miR-199a-3p, other miRNAs have also been found to function as major components in cancer biology. Additionally, miR-6886-3p was reported to play a role in hepatocellular carcinoma as a factor that decreases the levels of ubiquitin-specific peptidase 22 [48]. Finally, miR-374c-5p has also been reported to play a role in cancers such as breast cancer [49,50].

Thus, the functions of the most significant DE miRNAs identified herein have been studied previously; however, their clinical relevance has not been fully investigated. Therefore, our study evaluated the potential clinical applications of the top six DE miRNAs in terms of sensitivity, specificity, and AUC of the ROC curve. The results showed a sensitivity of 83.33–91.67% and a specificity of 60.00–90.00%. All of the top six DE miRNAs showed good ability to efficiently distinguish TB infections from HC with an AUC value greater than 0.85. The diagnostic values were high or comparable to the previously reported diagnostic values of other miRNAs. Qi et al., reported that the combination of miR-361-5p, miR-889, and miR-576-3p displayed strong potential for diagnostic utility with a high AUC value of 0.863 [51]. Additionally, previous studies have suggested that miR-30c and miR-142-3p serve as diagnostic biomarkers, with AUC values of 0.67 and 0.75, respectively [52]. Jia-Yi Cui et al., reported that AUC values of miR-769-5p, miR-320a, and miR-22-3p range from 0.69 to 0.97 in qRT-PCR validation [53].

Although sputum culture is a standard diagnostic tool for the treatment monitoring of TB, its results are not always indicative of successful treatment [54]. Thus, new biomarkers for predicting treatment response are needed for more effective management of patients with TB. In this study, we evaluated the diagnostic utility of the top six miRNAs in patients with TB who underwent anti-TB drug therapy by comparing the expression levels at time of diagnosis (T0) and after 2 months of intensive anti-TB drug therapy (T2); the results showed that the levels of miR-199b-3p, miR-199a-3p, miR-16-5p, and miR-374c-5p were significantly lower and had reverted to near baseline levels, i.e., to those of HCs. Similar to our findings in the present study, Wagh et al., reported a decrease in the level of miR-16 with treatment [46].

Because miRNAs regulate the expression of target genes to exert their functional features, the accurate prediction of target genes is important. Using miRNet, 108 potential target genes of miR-199b-3p, miR-199a-3p, miR-6886-3p, miR-6856-3p, miR-16-5p, and miR-374c-5p were identified. GO analysis of the identified target genes was performed using ClueGO software. The identified target genes are involved in the negative regulation of cellular amide metabolism, negative regulation of translation, transforming growth factor beta receptor signaling, intrinsic apoptotic signaling pathway, and negative regulation of cysteine-type endopeptidase activity.

This study has several limitations. First, the sample size was small. The expression of the identified DE miRNAs should be further investigated in a larger number of patients from multiple centers. Second, patients with other lung diseases were not included as controls for TB in this study. Further investigation with other lung disease controls is necessary to evaluate the ability of the DE miRNAs to discriminate TB from other lung diseases.

## 5. Conclusions

We suggest that miR-199b-3p, miR-199a-3p, miR-6886-3p, miR-6856-3p, miR-16-5p, and miR-374c-5p can offer promising diagnostic and treatment monitoring markers for TB patients.

## Figures and Tables

**Table 1:** Demographic and clinical characteristics of patients with active tuberculosis, individuals with latent tuberculosis infection, and healthy controls.

Characteristics	ATB (*n* = 12)	LTBI (*n* = 15)	HC (*n* = 10)	*p* Value
Gender (male, %)	8 (66.7%)	6 (40.0%)	6 (60.0%)	0.36
Age (mean ± SD)	41.0 ± 19.6	42.1 ± 15.1	34.4 ± 7.2	0.44
BCG scar (%)	9 (75.0%)	8 (53.3%)	9 (90.0%)	0.15
AFB stain positive	2 (16.7%)			
Culture positive	10 (83.3%)			
QFT-GIT result				
Positive	12 (100.0%)	15 (100.0%)	0 (0.0%)	
Intermediate	0 (0.0%)	0 (0.0%)	0 (0.0%)	
Negative	0 (0.0%)	0 (0.0%)	10 (100.0%)	

Abbreviations: ATB, active tuberculosis; LTBI, latent tuberculosis infection; HC, healthy control; QFT-GIT, QuantiFERON-TB gold in-tube assay.

**Table 2:** The diagnostic utility of differently expressed miRNAs for tuberculosis.

miRNAs	AUC (95% CI)	Cutoff	Sensitivity (%) (95% CI)	Specificity (%) (95% CI)	*p* Value
miR-199b-3p	0.96 (0.88–1.03)	>17.50	91.67 (61.52–99.79)	90.00% (55.50–99.75)	<0.001
miR-199a-3p	0.90 (0.78–1.03)	>48.00	83.33 (51.59–97.91)	90.00% (55.50–99.75)	<0.001
miR-6886-3p	0.88 (0.72–1.03)	>0.50	91.67 (61.52–99.79)	80.00% (44.39–97.48)	<0.001
miR-6856-3p	0.87 (0.73–1.02)	>0.50	91.67 (61.52–99.79)	60.00% (26.24–87.84)	<0.001
miR-16-5p	0.87 (0.71–1.02)	>2571.00	91.67 (61.52–99.79)	80.00% (44.39–97.48)	<0.001
miR-374c-5p	0.86 (0.71–1.01)	>24.50	83.33 (51.59–97.91)	80.00% (44.39–97.48)	<0.001

Abbreviations: AUC, area under the receiver operating characteristic curve; CI, confidence interval.

**Table 3:** List of gene ontology terms for predicted target genes of the top six differently expressed miRNAs.

GO ID	GO Terms	No. of Genes	*p* Value
GO:0034249	negative regulation of cellular amide metabolic process	9	<0.001
GO:0007179	transforming growth factor beta receptor signaling pathway	8	<0.001
GO:0017148	negative regulation of translation	8	<0.001
GO:2001242	regulation of intrinsic apoptotic signaling pathway	7	<0.001
GO:2000117	negative regulation of cysteine-type endopeptidase activity	7	<0.001
GO:0043154	negative regulation of cysteine-type endopeptidase activity involved in apoptotic process	6	<0.001
GO:0002576	platelet degranulation	6	<0.001
GO:0031091	platelet alpha granule	6	<0.001
GO:0001570	vasculogenesis	5	<0.001
GO:0003725	double-stranded RNA binding	5	<0.001
GO:0061014	positive regulation of mRNA catabolic process	4	<0.001
GO:0000289	nuclear-transcribed mRNA poly(A) tail shortening	4	<0.001
GO:1901983	regulation of protein acetylation	3	<0.001
GO:0060213	positive regulation of nuclear-transcribed mRNA poly(A) tail shortening	3	<0.001
GO:1900151	regulation of nuclear-transcribed mRNA catabolic process, deadenylation-dependent decay	3	<0.001
GO:1900153	positive regulation of nuclear-transcribed mRNA catabolic process, deadenylation-dependent decay	3	<0.001
GO:0060211	regulation of nuclear-transcribed mRNA poly(A) tail shortening	3	<0.001

Abbreviations: GO: gene ontology.

## Data Availability

The data generated or analyzed during this study are included in this published article and its additional files. Some of the datasets are available from the corresponding author upon reasonable request.

## Supplementary Materials

The following supporting information can be downloaded at: https://www.mdpi.com/article/10.3390/diagnostics12020369/s1, **Figure S1**: Validation of the expression levels of the selected miRNAs among study populations by qRT-PCR; **Table S1**: Demographic and clinical characteristics of patients with active tuberculosis, individuals with latent tuberculosis, and healthy control for validation of miRNA candidates; Table S2: Positive and negative results for the top six differentially expressed miRNAs; Table S3: The list of predicted target genes; Table S4: The GO analysis for the predicted targets.
