# Supplementary material for: Identification of MicroRNAs as Potential Blood-Based Biomarkers for Diagnosis and Therapeutic Monitoring of Active Tuberculosis
Source: Diagnostics (Basel). 2022 Feb 1;12(2):369. doi: 10.3390/diagnostics12020369 (PMC8871062; doi:10.3390/diagnostics12020369)
Supplement: Supplementary file 1 [file diagnostics-12-00369-s001.zip › diagnostics-Supplementary_data.pdf]

diagnostics

MDPI

Supplementary Data

Identification of MicroRNAs as Potential Blood-Based Biomarkers for Diagnosis and Therapeutic Monitoring of Active Tuberculosis

Junseong Kim [1,2,†], Heechul Park [1,2,†], Sung-Bae Park [1,2], Eun Ju Lee [1,2], Min-A Je [1,2], Eunsol Ahn [3], Bora Sim [4], Jiyoung Lee [1], Hyunwoo Jin [1,2], Kyung Eun Lee [1,2], Sang-Nae Cho [3,4], Young Ae Kang [5], Hyejon Lee [3,*], Sunghyun Kim [1,2,*] and Jungho Kim[1,*]

1 Department of Biomedical Laboratory Science, College of Health Sciences, Catholic University of Pusan, Busan 46252, Republic of Korea
2 Clinical Trial Specialist Program for In Vitro Diagnostics, Brain Busan 21 Plus Program, Graduate School, Catholic University of Pusan, Busan 46252, Republic of Korea
3 Clinical Vaccine Research Section, International Tuberculosis Research Center, Seoul 03772, Republic of Korea
4 Department of Microbiology, Institute of Immunology and Immunological Disease, Yonsei University College of Medicine, Seoul 03772, Republic of Korea
5 Division of Pulmonary and Critical Care Medicine, Department of Internal Medicine, Severance Hospital, Institute of Immunology and Immunological Disease, Yonsei University College of Medicine, Seoul 03772, Republic of Korea
† These authors contributed equally to this work.
* Correspondence: hyejonlee@gmail.com (H.L.); shkim0423@cup.ac.kr (S.K.); jutosa70@cup.ac.kr (J.K.). Tel.:+82-2-2228-0772 (H.L.); +82-51-510-0560 (S.K.); +82-51-510-0660 (J.K.). Fax: +82-2-313-7190 (H.L.); +82-51-510-0568 (S.K, J.K.)

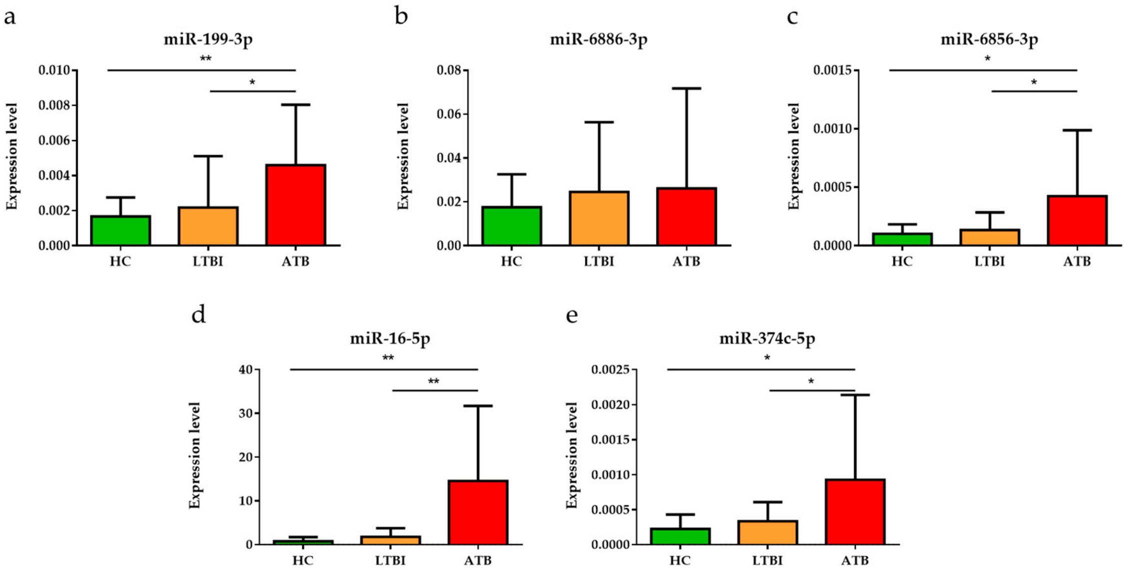

Figure S1. Validation of the expression levels of the selected miRNAs among study populations by qRT-PCR. The expression levels of (**a**) miR-199-3p (both miR-199a-3p and miR-199b-3p), (**b**) miR-6886-3p, (**c**) miR-6856-3p, (**d**) miR-16-5p, and (**e**) miR-374c-5p were measured in patients with active tuberculosis (ATB), individuals with latent tuberculosis infection (LTBI), and healthy controls (HCs). The data are shown as mean ± standard deviation. * $p < 0.05$, ** $p < 0.01$.
